# Ulceration of the Larynx

**Published:** 1844-10-19

**Authors:** 


#### ULCERATION OF THE LARYNX.

Mr. Bunn was consulted by a young man, who presented suspicious ulcers on the scalp: part of the glans penis sloughed, and the other part was destroyed by ulceration. He was more than once placed under the influence of mercury, but foul ulcers continued to appear about the root of the penis, and after a time he had cough, pain in the larynx, difficulty of breathing, etc. He died asphyxiated, and on examining the larynx, it was found extensively ulcerated, with exfoliated portions resembling carious bone, imbedded in loose, soft granulations.—*Ib.*

